# Exclusive breastfeeding practices in working women of Pakistan: A cross sectional study

**DOI:** 10.12669/pjms.335.12827

**Published:** 2017

**Authors:** Aroona Sabin, Farida Manzur, Saleem Adil

**Affiliations:** 1 Dr. Aroona Sabin, MBBS, M.S (Public Health). Senior Demonstrator, Department of Community Medicine, Aziz Fatima Medical & Dental College, Faisalabad, Pakistan; 2 Dr. Farida Manzur, MBBS, Diploma in Public Health. Professor and H.O.D, Department of Community Medicine, Aziz Fatima Medical & Dental College, Faisalabad, Pakistan; 3 Dr. Saleem Adil, MBBS, M.Sc. (Public Health). Assistant Professor, Department of Community Medicine, Aziz Fatima Medical & Dental College, Faisalabad, Pakistan

**Keywords:** Day-care facility, Exclusive breast feeding, Expression of breast milk, Working women

## Abstract

**Objective:**

To determine the prevalence of exclusive breast feeding in working women and to identify the factors effecting exclusive breast feeding in working women.

**Methods:**

This cross-sectional survey was conducted in Faisalabad city within a period of six months from June 2016 to December 2016. Working women of age 18 to 45 years, working as doctors, teachers, nurses and bankers in public (government) setup were included. The data was collected using interview method by pre-structured questionnaire. Multi-variable logistic regression model was developed considering EBF practice as dependent and the significant independent variables. Results were reported as Crude Odds Ratio (COR) or Adjusted Odds Ratio (AOR) with 95% Confidence Intervals (CIs).

**Results:**

Prevalence of exclusive breast feeding (EBF) was 166 (41.5%). EFB practice was significantly less in doctors and bankers as compared to nurses and teachers (p-value <0.001). Women working as nurses and teachers, having one or two children and short working hours had higher prevalence of exclusive breast feeding. Women having prior knowledge about EBF, training of EBF and women who had previously heard about EBF had five time higher rate of breast feeding. Women having knowledge of colostrum had three times higher EBF practice [odds ratio: 3.02 (1.86-4.91)]. Women having knowledge about hazards of bottle feeding had 12.7 times higher prevalence of EBF [odds ratio: 12.72 (5.70-28.38)]. Those who knew about expression of breast milk had three times higher prevalence of EBF [odds ratio: 3.0 (1.98-4.55)]. Mothers working in organizations that support EBF had 4.1 times higher prevalence of EBF [odds ratio: 4.1 (2.67-6.21)]. And proper training of mothers about correct expression technique of breast milk had 12 time [odds ratio: 12.06 (4.97-29.23)] higher prevalence of EBF.

**Conclusion:**

Long working hours, banking profession, family income and lack of proper knowledge about exclusive breast feeding are responsible for non-EBF practice in working women. Proper Knowledge and awareness about exclusive breastfeeding and provision of facilities for exclusive breastfeeding (EBF) by the organizations can play a significant role in promoting it.

## INTRODUCTION

Human milk is the most appropriate milk for human infants and uniquely adapted to the infant’s need.^1^ It is the best way of providing ideal nutrition for the healthy growth and development of infants.^2^ World Health Organization (WHO) has recommended that infants should be exclusively breastfed for the first six months of life to achieve optimal growth, development and health^3^ because exclusive breastfeeding in the first six months of life stimulates child’s immune systems and protects them from diarrhea and acute respiratory infections, two of the major causes of infant mortality in the developing world and improves their responses to vaccination.^4^ Avoiding colostrum and giving some pre-lacteal feed and bottle feeding are contributory factors for these preventable diseases which ultimately lead to high infant mortality.^5^ Exclusive breastfeeding during the initial months of life and continued breastfeeding through at least the first two year of life is associated with substantial reduction in the burden of infections and have many beneficial effects on mother’s health as well.^6^-^8^

According to UNICEF the global breastfeeding rates have remained stagnant since 1990 with only 36 per cent of children less than six months exclusively breastfed in 2012.^9^ According to demographic and health survey of Pakistan 2012-2013 rate of exclusive breastfeeding is 38% and 67% for non-exclusively breastfed.^10^ In a study conducted in Pakistan, the percentage of breastfeeding in housewives was 77% while only 23 % in working mothers.^11^ This clearly shows that a lot of women can be encouraged to exclusively breastfeed if they are properly supported to carry out this practice. So we conducted this study with the intention to find out the factors and barriers associated with exclusive breast feeding in working women of Pakistan.

## METHODS

This cross-sectional survey was conducted in Faisalabad city, one of the districts of Punjab province, Pakistan, within a period of six months from June 2016 to December 2016. Working women of age 18 to 45 years with 3 to 24 months postpartum who had initiated breastfeeding prior to the survey and returned to work at the time of the interview were included. Women working as doctors, teachers, nurses and bankers in public (government) setup were included. The hospitals chosen were Allied Hospital Faisalabad, District Headquarter Hospital Faisalabad, General hospital Faisalabad, Children Hospital Faisalabad and Faisalabad Institute of Cardiology. All the major government model schools situated within the city were selected for teachers. National Bank of Pakistan, Muslim Commercial Bank, United Bank Limited were the banks selected for bankers. Working mothers with any other co-morbidity and mothers with infants having any congenital abnormality were excluded.

### Data Collection

The data was collected using interview method by pre-structured questionnaire. The questionnaires had socio-demographic information of mothers, their knowledge and awareness on exclusive breastfeeding and factors that are in turn affecting their practices of exclusive breast feeding.

Working women were defined as the mothers 3 to 24 months postpartum, working in public sector including government hospitals, schools, colleges and banks. Exclusive breast feeding was defined according to the WHO definition, the practice of feeding breast milk only, including expressed breast milk, to infants and excluding water, other liquids, breast milk substitutes, and solid foods. Vitamin drops, minerals, oral rehydrating solution (ORS) and medicines may be given.^12^ Pre-lacteal feedings were defined as feedings that are given to infant other than mother’s milk before initiating breast feeding.^12^

### Data Analysis

The data was entered in statistical software (SPSS) version 20.0. Findings were presented in the form of tables and graphs. Univariate statistical tests were computed to identify all possible predictor variables. Then the multi-variable logistic regression model was developed considering EBF practice as dependent and the significant independent variables based on the result of the univariate test statistics. Results were reported as Crude Odds Ratio (COR) or Adjusted Odds Ratio (AOR) with 95 % Confidence Intervals (CIs).

## RESULTS

Four hundred (400) working mothers with infants less than six months of age were included in this study. Out of 400, 125 (31.25%) mothers were doctors, 125 (31.25%) were nurses, 100 (25.0%) were teachers and 50 (12.5%) mothers were bankers. Regarding monthly income, it was significantly high among doctors as compared to other professionals (p-value <0.001). Regarding support of family in going for work, 90.4% families of doctors were supportive, 95.2% in nurses, 96.0% in bankers and only 76.0% families of teachers were supportive. Support of families was less in women of teaching profession (p-value <0.001) (Table-I).

**Table-I:** Socio-demographic Characteristics of Study Participants.

	*Doctors*	*Nurses*	*Teachers*	*Bankers*	*P-value*
	***Age Distribution***				
< 20 Years	0.0 (0.0%)	14 (11.0%)	11 (11.0%)	0 (0.0%)	<0.001
20-35 Years	89 (71.2%)	54 (43.2%)	49 (49.0%)	18 (36.0%)	
> 35 Years	36 (28.8%)	57 (45.6%)	40 (40.0%)	32 (64%)	
	***Monthly Family Income***				
10-20 Thousands	0 (0.0%)	54 (43.2%)	24 (24.0%)	20 (40.0%)	<0.001
21-30 Thousands	0 (0.0%)	45 (36.0%)	61 (61.0%)	18 (36.0%)	
31-40 Thousands	34 (27.2%)	20 (16.0%)	12 (12.0%)	10 (20.0%	
> 40 Thousands	91 (72.8%)	6 (4.8%)	3 (3.0%)	2 (4.0%)	
	***Last Child’s Age***				
< 02 Months	37 (29.6%)	56 (44.8%)	19 (19.0%)	8 (16.0%)	<0.001
02-04 months	50 (40.0%)	25 (20.0%)	34 (34.0%)	24 (48.0%)	
4-6 months	38 (30.4%)	44 (35.2%)	47 (47.0%)	18 (36.0%)	
	***Number of Children***				
1	59 (47.2%)	38 (30.4%)	22 (22.0%)	14 (28.0%)	<0.001
2	19 (15.2%)	39 (31.2%)	33 (33.0%)	30 (60.0%)	
3	32 (25.6%)	25 (20.0%)	17 (17.0%)	2 (4.0%)	
> 4	15 (12.0%)	23 (18.4%)	28 (28.0%)	4 (8.0%)	
	***Mode of Normal Delivery***				
Normal Vaginal	23 (18.4%)	30 (24.0%)	18 (18.0%)	4 (8.0%)	0.04
Assisted Vaginal	72 (57.6%)	59 (47.2%)	45 (45.0%)	34 (68.0%)	
C-section	30 (24.0%)	36 (28.8%)	37 (37.0%)	12 (24.0%)	
	***Daily Working Hours***				
06 hours	21 (16.8%)	111 (88.8%)	89 (89.0%)	0 (0.0%)	<0.001
08 hours	47 (37.6%)	14 (11.2%)	11 (11.0%)	44 (88.0%)	
10 hours	49 (39.2%)	0 (0.0%)	0 (0.0%)	6 (12.0%)	
>10 hours	8 (6.4%)	0 (0.0%)	0 (0.0%)	0 (0.0%)	
	***Family Support in Going for Working***				
Yes	113 (90.4%)	119 (95.2%)	76 (76.0%)	46 (96.0%)	<0.001
No	12 (9.6%)	6 (4.8%)	24 (24.0%)	4 (8.0%)	

**Fig.1:**
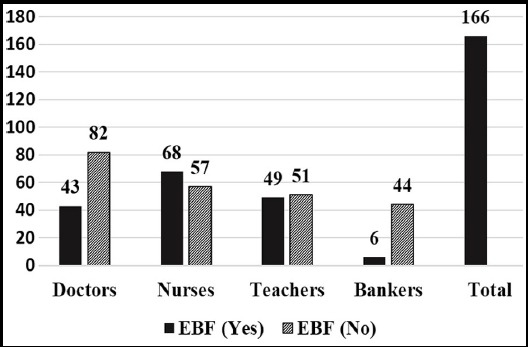
Practice of Exclusive Breast Feeding (EBF) among Working Mothers.

Prevalence of exclusive breast feeding (EBF) was 166 (41.5%). However there was significant difference in practice of exclusive breast feeding among professions. EFB practice was significantly less in doctors and bankers as compared to nurses and teachers (p-value <0.001) (Fig.1).

Regarding association of socio-demographic factors with practices of EBF, there was a significant influence of nursing and teaching profession in increasing the practice of EBF. Having less number of children (e.g. 1 to 2) was associated with increased prevalence of EBF among working mothers. Longer working hours have negative influence on EBF (p-value <0.001) (Table-II).

**Table-II:** Association of socio-demographic characteristics with exclusive breast feeding.

	*EBF (n=166)*	*Non-EBF (n=234)*	*Odds Ratio (95% CI)*	*P-value*
***Age***				
< 20 Years	13 (7.8)	12 (5.1)	0.57 (0.12-2.65)	0.50
21-35 Years	87 (52.4)	123 (52.6)	0.34 (0.12-0.91)	
> 35 Years	66 (39.8)	99 (42.3)	1
***Working Profession***				
Doctor	43 (25.9)	82 (35.0)	3.18 (0.60-16.97)	<0.001
Nurse	68 (41.0)	57 (24.4)	27.94 (6.67-117.0)	
Teacher	49 (29.5)	51 (21.8)	40.04 (8.86-180.95)	
Banker	6 (3.6%)	44 (18.8)	1	
***Monthly Family Income***				
10-20 Thousands	41 (24.7)	57 (24.4)	0.16 (0.03-72)	0.02
21-30 Thousands	52 (31.3)	72 (30.8)	0.18 (0.05-0.68)	
31-40 Thousands	41 (24.7)	35 (15.0)	2.55 (1.14-5.72)	
> 40 Thousands	32 (19.3)	70 (39.9)	1	
***Number of Children***				
1	51 (30.7)	82 (35.0)	8.0 (1.42-44.78)	0.02
2	58 (34.9)	63 (26.9)	18.32 (4.27-78.46)	
3	33 (19.9)	43 (18.4)	6.60 (2.19-19.87)	
> 4	24 (14.5)	46 (19.7)	1	
***Mode of Delivery***				
Normal Vaginal	29 (17.5)	46 (19.7)	0.79 (0.83-3.42)	0.73
Assisted Vaginal	91 (54.8)	119 (50.9)	1.93 (0.60-6.12)	
C-section	46 (27.7)	69 (29.5)	1	
***Daily Working Hours***				
06 hours	111 (66.9)	110 (47.0)	0.18 (0.02-1.6)	0.001
08 hours	38 (22.9)	78 (33.3)	0.23 (0.03-1.72)	
10 hours	14 (8.4)	41 (7.5)	0.16 (0.02-1.05)	
> 10 hours	3 (1.8)	5 (2.1)	1	
***Family Support***				
Yes	147 (88.6)	207 (88.5)	1.00 (0.54-1.88)	0.98
No	19 (11.4)	27 (11.5)		

As regards Knowledge and Awareness of EBF with EBF practice, prior knowledge of participants about exclusive breast feeding and training of exclusive breast feeding with EBF, Mothers who had previously heard about EBF had five time higher rate of breast feeding as compared to mothers who had never heard about EBF. Women having knowledge of colostrum, mothers who personally thought that they can continue breast feeding along with work, women who know about hazards of bottle feeding had significantly higher prevalence of EBF as compared to mothers who do not know. Women having knowledge about expression of breast milk have three times higher prevalence of EBF [odds ratio: 3.0 (1.98-4.55)]. Mothers who thought that a supportive working environment can have significant influence in promoting the practice of EBF have 2.9 times higher prevalence of EBF [2.90 (1.30-6.51)] (Table-III).

**Table-III:** Association of knowledge of EBF with exclusive breast feeding.

	*EBF (n=166)*	*Non-EBF (n=234)*	*Odds Ratio (95% CI)*	*P-value*
***Have you heard about EBF practices?***				
Yes	157 (94.6)	176 (75.2)	5.75 (2.76-11.98)	<0.001
No	9 (5.4)	58 (24.8)		
***Have you received any training/guidance about EBF practices?***				
Yes	45 (27.1)	39 (16.6)	1.86 (1.14-3.02)
No	121 (72.9)	195 (83.4)		
***Do you know about the importance of colostrum?***				
Yes	138 (83.1)	145 (62.0)	3.02 (1.86-4.91)	<0.001
No	28 (16.9)	89 (38.0)		
***When should BF be started?***				
Within 1 hour	141 (84.9)	169 (72.2)	1.67 (0.15-18.59)	0.013
Within 12 hours	15 (9.0)	49 (20.9)	0.61 (0.05-7.23)	
Within 24 hours	9 (5.4)	14 (6.0)	1.29 (0.10-16.34)	
> 24 hours	1 (0.6)	2 (0.9)	1	
***Have you been counseled about the hazards of bottle feeding?***				
Yes	159 (95.8)	150 (64.1)	12.72 (5.70-28.38)	0.001
No	7 (7.7)	84 (35.9)		
***Do you think bottle feeding is easier to practice for working mothers?***				
Yes	146 (88.0)	231 (98.7)	0.09 (0.03-0.32)	<0.001
No	20 (12.0)	3 (1.3)		
***Should pre-lacteal feeds ‘GHURTI’ can be given to a newborn***				
Yes	57 (34.3)	108 (46.2)	1.57 (0.92-2.68	0.06
No	70 (42.2)	79 (33.8)	0.94 (0.55-1.60)	
Don’t Know	39 (23.5)	47 (20.1)	1	
***Have you heard about expression of breast milk?***				
Yes	111 (66.9)	94 (40.2)	3.0 (1.98-4.55)	<0.001
No	55 (33.1)	140 (59.8)		
***Is Expressed Milk Beneficial for Working Mothers?***				
Yes	99 (84.6)	71 (71.8)	2.17 (1.11-4.22)	0.02
No	18 (15.4)	29 (28.3)
***Do you think EBF is practically possible with work?***				
Yes	98 (59.0)	91 (38.9)	2.26 (1.51-3.40)	<0.001
No	68 (41.0)	143 (61.1)		
***Do you think our Medical practitioners’ plays a positive role in promoting EBF?***				
Yes	121 (72.9)	150 (64.1)	1.50 (0.97-2.32)	0.06
No	45 (27.1)	84 (35.9)		
***Do you feel comfortable while breast feeding at your work place?***				
Yes	50 (30.1)	86 (36.8)	0.74 (0.48-1.13)	0.17
No	116 (69.9)	148 (63.2)		
***Do you think that a supportive workplace environment can promote EBF?***				
Yes	158 (95.2)	204 (87.2)	2.90 (1.30-6.51)	0.007
No	08 (4.8)	30 (12.8)		
***Do you think provision of day care center at work place can benefit EBF practices?***				
Yes	162 (97.6)	223 (95.3)	1.99 (0.62-6.38	0.23
No	4 (2.4)	11 (4.7)		
***Do you think longer maternity leave can increase the successful practice of EBF?***				
Yes	134 (80.7)	215 (91.9)	0.37 (0.20-0.68)	0.001
No	32 (19.3)	19 (8.1)		

Regarding association of facilities provided by Organization to mothers after child birth, decreasing the working hours after maternity leaves, mothers working in organization that support for EBF and mothers working in organizations having day care facility had higher prevalence of EBF. Proper training of mothers about correct expression technique of breast milk had 12 time [odds ratio: 12.06 (4.97-29.23)] higher prevalence of EBF as compared to mother who did not get training of correct expression technique (Table-IV).

**Table-IV:** Association of facilities given by organization to working mothers for exclusive breast feeding.

	*EBF (n=166)*	*Non-EBF (n=234)*	*Odds Ratio (95% CI)*	*P-value*
***Duration of maternity leave***				
3 months	150 (90.4)	216 (92.3)	0.57 (0.17-1.93)	0.65
6 months	10 (6.0)	13 (5.6)	0.64 (0.15-2.72)	
More than 6 months	6 (3.6)	5 (2.1)	1	
***Decrease working hours after maternity***				
Yes	59 (35.5)	56 (23.9)	1.75 (1.13-2.71)	0.01
No	107 (64.5)	178 (76.1)		
***Work place support regarding EBF practice.***				
Yes	103 (62.0)	67 (28.6)	4.1 (2.67-6.21)	<0.001
No	63 (38.0)	167 (71.4)		
***Day-Care Facility***				
Yes	79 (47.6)	75 (32.1)	1.92 (1.27-2.90)	0.002
No	87 (52.4)	159 (67.9)		
***Training about the correct expression technique***				
Yes	40 (24.1)	6 (2.6)	12.06 (4.97-29.23)	<0.001
No	126 (75.9)	228 (97.4)		

## DISCUSSION

In Pakistan, infant mortality rate is very high malnutrition and infections are most common cause of infant mortality and morbidity. Breast-milk prevents the newborn from malnutrition and helps to increase the immunity. So Breast feeding is very crucial for the proper growth of newborn especially for the first 6 months of life.^13^

In this study, we evaluated the prevalence of exclusive breast feeding (EBF) among working women of Faisalabad. We evaluated their socio-demographic characteristics, their knowledge about awareness and training of EBF and provision of facilities for EBF in working organization and evaluated their association with EBF. We chose cross-sectional study design for this study because cross-sectional design provides easy calculation of prevalence of many factors at a single point in time. We took women working in four different professions e.g. doctors, nurses, teachers and bankers. In our study, the prevalence of EBF in children under 6 months of age was 41.5%.

In our study, working profession, monthly family income, number of children and duration of working hours were main socio-demographic factors that interfere with exclusive breastfeeding.

Mother’s knowledge about EBF, training of exclusive breast feeding, importance of colostrum feed, timing to start breastfeeding and knowledge about hazards of bottle feeding and knowledge about expressed milk was significant factors in increasing the prevalence of EBF. Mother’s personal perceptions mother’s personal perceptions about continuing breastfeeding with job and provision of supportive working environment were positively associated with EBF practices. While the perception that longer maternity leaves can increase the prevalence of EBF and it is easier to practice bottle feeding as compared to breastfeeding in working women were negatively associated and were responsible for decreasing the prevalence of EBF.

In a local study, conducted in Bahawalpur City of Pakistan by Knechi et al.^14^, the prevalence of breast feeding was 30.0% and only 25.0% in children with age less than 6 months. According to Afzal et al.^15^ most of the mothers in Pakistan have knowledge about benefits of breast feeding but these women have some other believes that interfere with the practice of EBF. Kulsoom et al.^16^ found that poverty, illiteracy and female child are significantly associated with termination of EBF in Pakistani mothers.

According to study by Aslam et al.^17^ conducted in Gilgit, Pakistan, 1^st^ child is deprived of EBF in many cases and most common reasons for non-EBF in 1^st^ child were low socioeconomic status and gender biasedness. According to that study, prevalence of EBF was high in illiterate mothers.

According to Farrukh et al. prevalence of early failure of exclusive breast feeding was 41.9%.^18^ Lack of proper knowledge about benefits of EBF and less amount of breast milk were two main causes of early failure of EBF.

Yaqoob et al.^19^ concluded that illness of mother or child, inadequate production of breast milk and working mothers ware common risk factors of failure of EBF in Islamabad population.

Data from another study conducted in Ghana also showed that although awareness on exclusive breast feeding among professional working mothers is almost universal (99 %), the practice of EBF at six months is low (10.3 %).^20^ Elsewhere, Al-binali^21^ found 89 % of mothers had a good knowledge about exclusive breastfeeding but only a small percentage (8.3%) engaged in the practice for the first six months. The same results can be drawn from our study which also shows higher level of knowledge of EBF (333 out of 400 women) but low practice of EBF (166 out of 400 women).

Data from studies conducted in Nigeria and United States showed that the main source of education about breastfeeding provided to working mothers is the health worker and the medical practitioners.^22^,^23^ These results also support the findings of this study that the level of guidance and training regarding EBF had a positive influence on EBF practices.

Hassan et al. also proposed that all working mothers had a good knowledge about exclusive breastfeeding and the importance of breastfeeding infants for the first six months. But the level of practice was not up to that. These authors suggested that the improved educational status and increased knowledge among working mothers can contribute a lot towards workplace lactation.^24^

In our study, 362 out of 400 women thought that supportive work place can benefit EBF practices at birth. While 385 out of 400 were of the view that provision of day care has a positive influence on EBF. To get flexible working timings can also contribute to increased breastfeeding in working women. Heymann et al., found that globally the rate of exclusive breastfeeding of children under 6 months of age was 9% greater in countries that assured paid breastfeeding breaks at workplace and vice versa.^25^ A qualitative study in Pakistan, also found that flexible schedule at workplace of mothers that are breastfeeding was very important for sustaining breastfeeding in working women.^26^

## CONCLUSION

Long working hours, banking profession, family income and lack of proper knowledge about exclusive breast feeding are responsible for non-EBF practice in working women. Proper knowledge and awareness about exclusive breastfeeding and provision of facilities for exclusive breastfeeding (EBF) by the organizations can play a significant role in increasing the prevalence of EBF.

## RECOMMENDATIONS

The study determined that majority of the working mothers did not consider their work place environment comfortable and supportive for breast feeding. Therefore, interventions should be encouraged to incorporate helpful strategies regarding breast feeding in all the working organizations including female staff to promote exclusive breastfeeding practices in working mothers, so that it can contribute towards reducing infant morbidity and mortality rates. Continuous evaluation of evidence based implementation practices, can effectively improve exclusive breastfeeding outcomes, meeting individual and population needs at the community level.

### Authors’ Contribution

**AS:** Conceived, designed the research methodology, wrote the manuscript and is accountable for originality of this research work.

**FM:** Supervised the research project, did final approval of the manuscript.

**SA:** Helped in data collection, analysis.
